# *GSTM1* and *GSTT1* polymorphisms contribute to renal cell carcinoma risk: evidence from an updated meta-analysis

**DOI:** 10.1038/srep17971

**Published:** 2015-12-14

**Authors:** Wentao Huang, Hua Shi, Qi Hou, Zu Mo, Xiangwei Xie

**Affiliations:** 1 Department of Urology, Third Affiliated Hospital, Sun Yat-sen University, Guangzhou 510630, China; 2 Department of Urology, Guangzhou First People’s Hospital, Guangzhou Medical University, Guangzhou 510180, China; 3 Department of Urology, Longgang District Central Hospital, Shenzhen 518116, China

## Abstract

Emerging evidences suggest that *GSTM1* and *GSTT1* are involved in the detoxification of carcinogens, and polymorphisms in this gene that result in a loss of enzyme activity may increase the risk of renal cell carcinoma (RCC). Thus, to evaluate the association of *GSTM1* and *GSTT1* polymorphisms and RCC, we performed an updated meta-analysis of 10 case-control studies by RevMan 5.2, and the publication bias was tested using STATA 11.0. The meta-analysis showed that the single locus *GSTM1* and *GSTT1* polymorphisms were not significantly associated with a risk of RCC in a recessive model. However, that wild-type genotype versus the dual null genotype of *GSTM1-GSTT1* showed a positive association with RCC risk (OR = 0.70; 95% CI = 0.51–0.98; *P *= 0.04). In another analysis of subjects exposed to pesticides, we found that the *GSTM1* wild-type genotype was associated with increased RCC risk in Europeans (OR = 2.72; 95% CI = 1.54–4.82; *P *= 0.0006). We also identified an association between the *GSTT1* wild-type and lower RCC TNM staging (I + II versus III + IV: OR = 1.88; 95% CI = 1.09–3.26; *P *= 0.02). This meta-analysis suggests that there may be a relationship between the *GSTM1* and *GSTT1* wild-type genotype and RCC.

Renal cell carcinoma (RCC) is the predominant form of kidney malignancy (approximately 90% of all cases) and one of the leading causes of global cancer death. Approximately 300,000 people were diagnosed with kidney cancer in 2008, and 100,000 people died from the disease^1^. An estimated 63,920 Americans will be diagnosed with renal cancer and 13,860 will die of the disease in the United States in 2014^2^. The morbidity and mortality rates are approximately twice as high for males as for females. The incidence of RCC is increasing worldwide, with the highest incidence occurring in developed countries, especially in Europe, North America, and Australia^3^,^4^.

It is well-known that exposure to potential carcinogens is an etiologic factor for RCC^5^. Glutathione S-transferases (GSTs) are a superfamily of enzymes that are subdivided into 7 classes (α, μ, ω, π, σ, θ, ξ)^6^, and are known to protect cells by catalyzing the detoxification of electrophilic compounds, including exogenous products (carcinogens, therapeutic drugs, environmental toxins) and endogenous oxidative products, through conjugation with glutathione^7^. This conjugation reaction is an important step of detoxification and facilitates their excretion from the body, thereby decreasing the associated toxicity. Considering the damage to DNA induced by electrophilic compounds, GSTs are important for maintaining genomic integrity. Therefore, GST enzymes may potentially play a key role in the development of RCC.

GSTM1 and GSTT1 are the most frequently studied enzymes in the GST family. The *GSTM1* gene encodes the GST-μ1 enzyme and has been mapped to chromosome 1p13.3. There are two variants of *GSTM1* that can occur through a substitution and a deletion, respectively. The substitution polymorphism changes C-to-G at base position 534, resulting in a lysine-to-asparagine switch, which does not appear to affect enzyme function. The deletion variant is a null genotype of *GSTM1*, which is more commonly studied than the substitution variant because it leads to the absence of GST-μ1 synthesis^6^. In this article, we only studied the deletion variant of *GSTM1*. The *GSTT* gene encodes the GST-θ subfamily, which is located at chromosome 22q11.2 and consists of two genes, *GSTT1* and *GSTT2*. The duplicated *GSTT2* is a pseudogene (named GSTT2P). The *GSTT1* gene is embedded in a region with extensive homologies and flanked by two 18 kb regions, which were identified as deletion/junction regions of the *GSTT1* null allele^8^. Moreover, the deletion variant of *GSTT1* is a null genotype, which occurs less frequently and results in complete absence of the enzyme.

To date, a limited number of molecular epidemiological studies have investigated the association between the *GSTM1* and *GSTT1* polymorphisms and RCC, and the conclusions have not been consistent^9^,^10^,^11^,^12^,^13^,^14^,^15^,^16^,^17^,^18^. A single case-control study may fail to demonstrate this complicated genetic relationship due to the small sample size, and thus a meta-analysis could increase the statistical power for detecting overall effects. Recently, a few meta-analyses^19^,^20^,^21^ have attempted to uncover the relationship between the GSTM1 and GSTT1 polymorphisms and RCC. However, Liu *et al.*^20^ did not investigate the relationship between *GSTT1* polymorphisms and RCC and the relationship between the combination of *GSTM1* and *GSTT1* polymorphisms and RCC. Cheng *et al.*^19^ also studied the association between *GSTM1* and *GSTT1* polymorphisms and RCC, but the data extracted from the studies by Longuemaux *et al.* and De Martino *et al.* were controversial and the data from the studies by Salinas *et al.* and Buzio *et al.* were not included. In addition, Yang *et al.*^21^ incorrectly extracted duplicate genetic information from the studies by Wiesenhütter and Brüning, as the RCC case and control groups in Wiesenhütter *et al.* were comprised of case and control groups from Brüning *et al.* In this study, we conducted an updated meta-analysis on all currently available to validate the further relationship between the *GSTM1* and *GSTT1* polymorphisms and RCC.

## Results

### Description of studies

**Figure 1:**
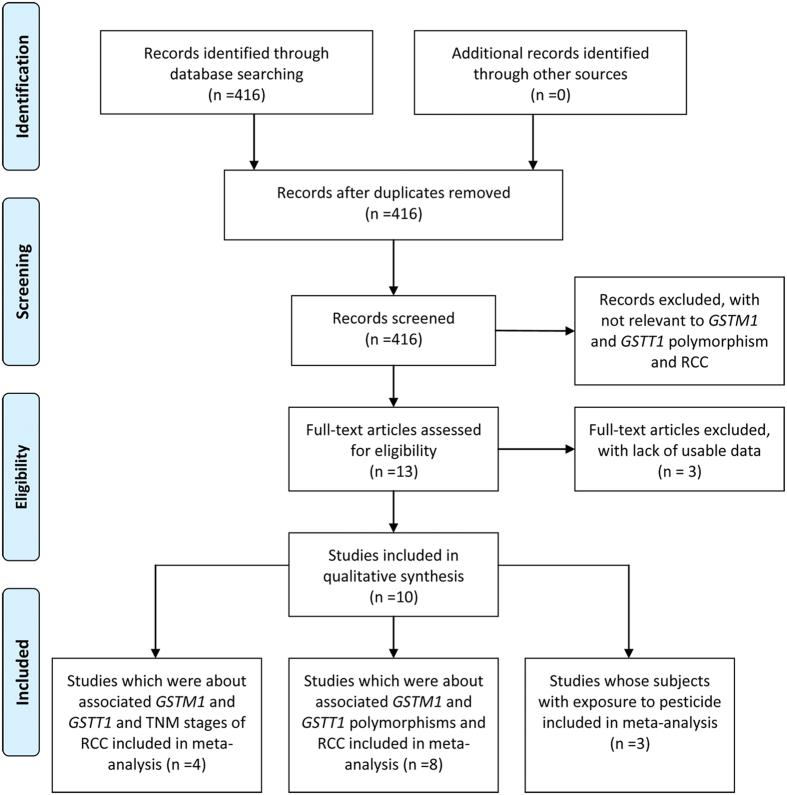
Flow diagram of study identification.

A total of 416 articles were identified through a literature search in PubMed, ISI, Wangfang, and CNKI databases. Ten eligible studies were retrieved for detailed evaluation (Fig. 1). We included eight studies that described an association between GSTM1 and RCC by comparing RCC with healthy controls, which included 1826 cases and 3377 controls. In addition, there were 1831 cases and 3407 controls in the same eight studies regarding GSTT1 and RCC (Table 1). Five of the studies were conducted in Europe, and three studies were conducted in America, Australia, and India, respectively. Four of the eight studies described an association between the dual null genotype of GSTM1 and GSTT1 and risk of RCC, which included 1307 cases and 2057 controls. Moreover, three studies, all conducted in Europe, assessed the association between GSTM1 or GSTT1 and RCC in patients exposed to pesticide or trichloroethene, which both included 107 cases and 101 controls (Table 2). Three studies (2 in Europe, 1 in India) explored the association between GSTM1 and RCC staging, including 224 GSTM1 wild-type patients and 247 GSTM1 null patients. In addition, four studies (3 in Europe, 1 in India) explored the association between GSTT1 and RCC staging, including 335 GSTT1 wild-type patients and 251 GSTT1 null patients (Table 3). The GSTM1 and GSTT1 polymorphisms were detected by PCR in all of the studies.

## Meta-analysis results

### Association between *GSTM1* and *GSTT1* polymorphisms and RCC risk^9^,^10^,^11^,^12^,^13^,^14^,^15^,^16^

**Figure 2:**
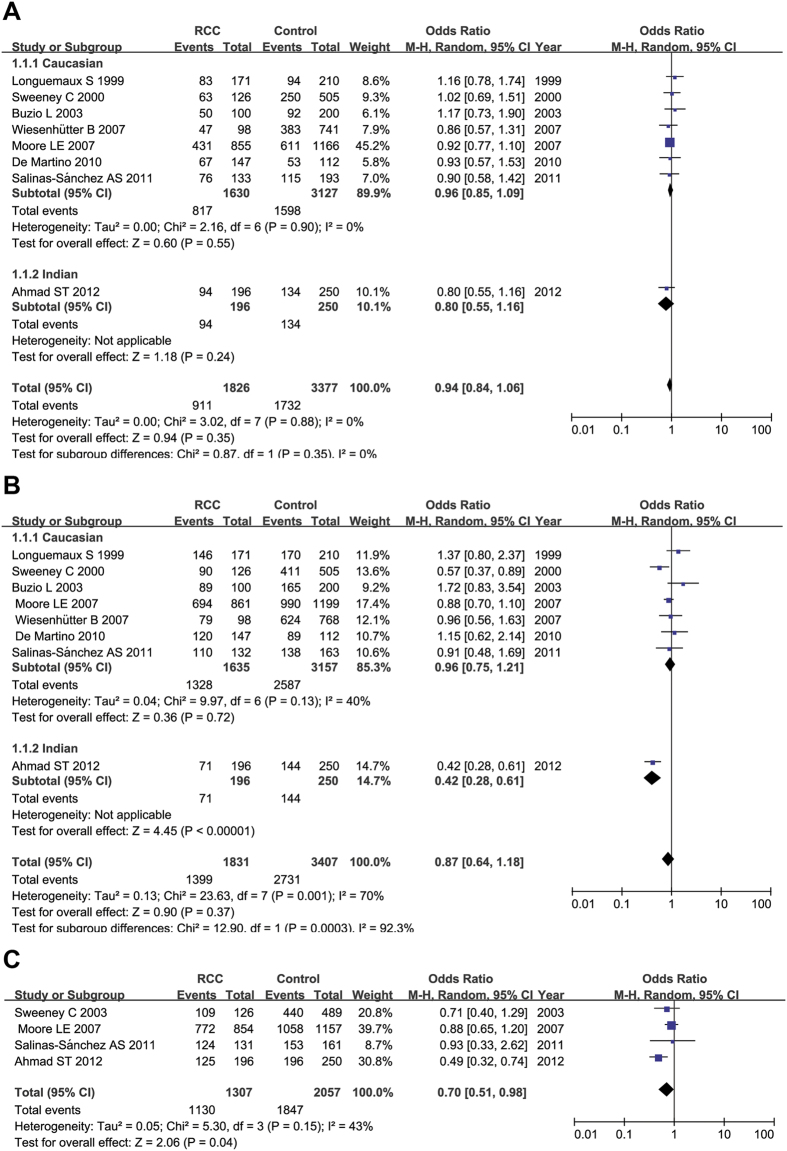
Forest plots describing the association of *GSTM1* and *GSTT1* wild-type genotype with RCC risk. (**A**) The association of *GSTM1* wild-type genotype with RCC risk. (**B**) The association of *GSTT1* wild-type genotype with RCC risk. (**C**) The association of combination of *GSTM1* and *GSTT1* polymorphism with RCC. OR: odds ratio; CI: confidence interval; I^2^, measure to quantify the degree of heterogeneity in meta-analysis.

The results of the meta-analysis did not find any association between *GSTM1* polymorphisms and RCC susceptibility for the recessive model (for present/present + present/null versus null/null: OR = 0.94, 95% CI = 0.84–1.06; *P *= 0.35; I^2^ = 0%, 95% CI = 0%–45%, τ^2^ = 0.00). After excluding the study conducted by Ahmad *et al.* because it did not focus on Caucasians, the results of the meta-analysis also did not indicate any association between the *GSTM1* polymorphism and RCC susceptibility for the recessive model in Caucasians (for present/present + present/null versus null/null: OR = 0.96, 95%CI = 0.85–1.09; *P* = 0.55; I^2^ = 0%, 95% CI = 0%–45%, τ^2^ = 0.00) (Fig. 2A). We also did not find any association between the *GSTT1* polymorphism and RCC susceptibility for the recessive model (for present/present+ present/null versus null/null: OR = 0.87, 95% CI = 0.64–1.18; *P* = 0.37; I^2^ = 70%, 95% CI = 38%–86%, τ^2^ = 0.13). After excluding Ahmad *et al.*, the results of the meta-analysis also did not show any association between *GSTT1* polymorphisms and RCC susceptibility for the recessive model in Caucasians (for present/present + present/null versus null/null: OR = 0.96, 95% CI = 0.75–1.21; *P *= 0.72; I^2^ = 40%, 95% CI = 0%–75%, τ^2^ = 0.04) (Fig. 2B). However, the association between the combination of *GSTM1* and *GSTT1* polymorphisms and risk of RCC for the recessive model (for *GSTM1*(present)/*GSTT1*(present) + *GSTM1*(present)/*GSTT1*(null) *GSTM1*(null)/*GSTT1*(present) versus *GSTM1*(null)/*GSTT1*(null): OR = 0.70; 95 %CI = 0.51–0.98; *P *= 0.04; I^2^ = 43%, 95% CI = 0–81%, τ^2^ = 0.05) was statistically significant (Fig. 2C).

### Association between *GSTM1* and *GSTT1* polymorphisms and RCC risk of subjects exposed to occupational pesticides^16^,^17^,^18^

**Figure 3:**
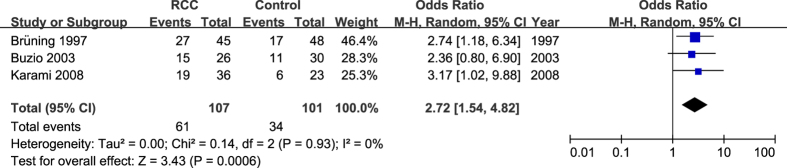
Forest plots describing the association of *GSTM1* wild-type genotype with RCC under the condition of all subjects with exposure to pesticides. OR: odds ratio; CI: confidence interval; I^2^, measure to quantify the degree of heterogeneity in meta-analysis.

In studies that assessed subjects exposed to pesticides, we found that *GSTM1* wild-type was significantly associated with increased RCC risk (present/present+ present/null versus null/null: OR = 2.72, 95% CI = 1.54–4.82; *P *= 0.0006; I^2^ = 0%, 95%CI = 0–89%, τ^2^ = 0.00) (Fig. 3). However, we did not find an association between *GSTT1* wild-type and RCC (present/present + present/null versus null/null: OR = 1.83, 95% CI = 0.76–4.38; *P* = 0.18; I^2^ = 31%, 95% CI = 0–76%,τ^2^ = 0.18).

### Association between *GSTM1* and *GSTT1* polymorphisms and clinical TNM stages of RCC^11^,^12^,^13^,^14^

**Figure 4:**
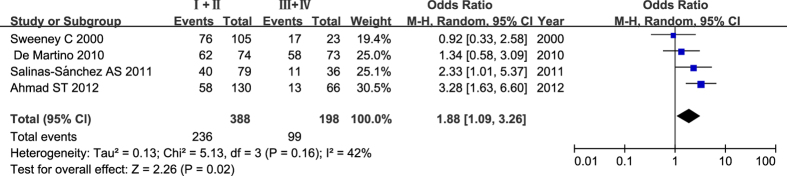
Forest plots describing the association of *GSTT1* wild-type genotype with RCC TNM stages (recessive model in stage I + II versus stage III + IV). OR: odds ratio; CI: confidence interval; I^2^, measure to quantify the degree of heterogeneity in meta-analysis.

We performed a quantitative assessment of OR of wild-type and null genotype frequency in each TNM stage from the included studies, which assessed the association between *GSTM1* and *GSTT1* polymorphisms and TNM stages of RCC. The results did not show any significant difference for *GSTM1* wild-type (recessive model in stage I versus stage II + III + IV: OR = 1.24, 95% CI = 0.63–2.43; *P *= 0.54; I^2^ = 76%, 95% CI = 19–92%, τ^2^ = 0.27; recessive model in stage I +  II versus stage III + IV: OR = 1.71, 95% CI = 0.49–6.01; *P *= 0.40; I^2^ = 89%, 95% CI = 68–96%, τ^2^ = 1.08). However, subjects with *GSTT1* wild-type showed an association with lower clinical TNM stages of RCC (recessive model in stage I versus stage II + III + IV: OR = 1.58, 95% CI = 0.89–2.79; *P* = 0.12; I^2^ = 58%, 95% CI = 0%-86%, τ^2^ = 0.19; recessive model in stage I + II versus stage III +  + IV: OR = 1.88, 95% CI = 1.09–3.26; *P* = 0.02; I^2^ = 42%, 95% CI = 0–80%, τ^2^ = 0.13) (Fig. 4).

### Publication bias

**Figure 5:**
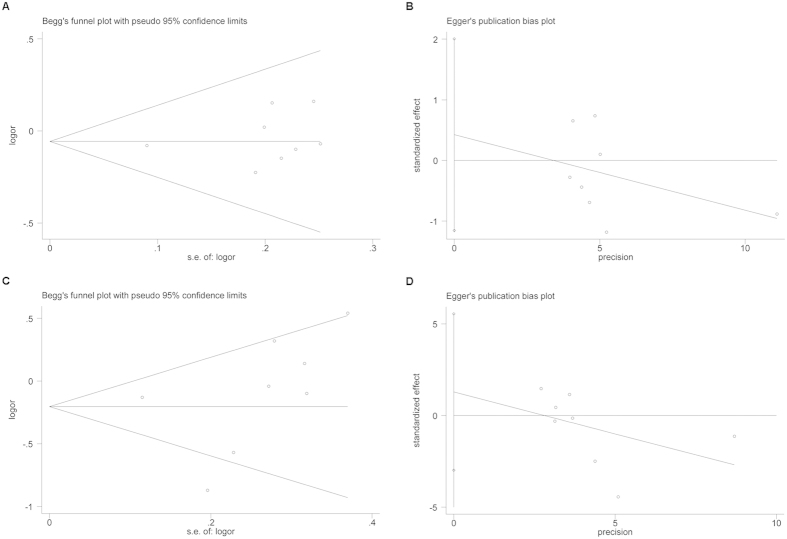
Publication bias of *GSTM1* and *GSTT1* polymorphism and RCC risk. (**A**) Begg’s publication bias of *GSTM1* polymorphism and RCC risk (*P *= 0.322). (**B**) Egger’s publication bias of *GSTM1* polymorphism and RCC risk (*P *= 0.536). (**C**) Begg’s publication bias of *GSTT1* polymorphism and RCC risk (*P *= 0.138). (**D**) Egger’s publication bias of *GSTT1* polymorphism and RCC risk (*P *= 0.490).

The Begg’s rank correlation method and Egger’s weighted regression method were used to assess publication bias. Because of the number of studies included in the analysis, we only assessed publication bias for the association between *GSTM1* and RCC risk (Begg’s test: *P* = 0.322; Egger’s test: *P* = 0.536), and *GSTT1* and RCC (Begg’s test: *P *= 0.138; Egger’s test: *P* = 0.490). No evidence of publication bias was observed in our meta-analysis of the eight studies (Fig. 5).

## Discussion

The identification of SNPs that affect gene function or expression and contribute to RCC susceptibility play a critical role in helping predict individual and population risk and understanding the pathology of RCC. Deletion variants have been identified in the *GSTM1* and *GSTT1*, which are enzymes that play important roles in phase II detoxification and for maintaining genomic integrity. The *GSTM1* and *GSTT1* null genotype have been hypothesized to be associated with increased risk for RCC due to reduced protection against endogenous reactive oxidants. The *GSTM1* and *GSTT1* null polymorphism are associated with lung cancer^22^, prostate cancer^23^, hepatocellular carcinoma^24^, and head and neck squamous cell carcinoma^25^. However, there are controversies in the relationship between the *GSTM1* and *GSTT1* polymorphisms and RCC susceptibility. In 1999, Longuemaux *et al.* first investigated the correlation between the *GSTM1* null polymorphisms and RCC, but his and followed studies did not identify a significant correlation^9^,^10^,^11^. With respect to the *GSTT1* genotype, Sweeney *et al.* and Ahmad *et al.* found it to be associated with an increased risk of RCC, but other studies did not reach the same conclusion. In this meta-analysis, we failed to identify an association between the single locus *GSTM1* and *GSTT1* polymorphisms and increased risk of RCC. However, with regard to the dual null genotype of *GSTM1* and *GSTT1*, we found this genotype to be significantly associated with an increased risk of RCC. This finding indicates that the protective effects provided by only one of the *GSTM1* and *GSTT1* wild-type genes are minor, but cumulative. However, when both *GSTM1* and *GSTT1* genes are deleted, there would be significant loss of detoxification and increased risk of RCC.

In our meta-analysis of the association between the *GSTM1* and *GSTT1* wild-type genotype and RCC risk of subjects exposed to pesticides, we included a history of trichloroethene exposure, because it is widely used in industrial and agriculture processes, especially in the menstruum of pesticides. In contrast to previous results, we found that subjects exposed to occupational pesticides with the *GSTM1* wild-type genotype are significantly associated with an increased risk of RCC. However, the *GSTT1* wild-type genotype did not have a significant relationship with RCC risk. These results support the hypothesis that *GSTM1* might play a role in the interaction with environmental factors, especially occupational exposure to toxic compounds. However, because these results conflict with previous findings, the role of *GSTM1* in RCC remains unclear at this time.

Fortunately, some current studies have provided valuable clues that could help us decipher this puzzle. The biological mechanism of our findings may involve unique biochemical processes in the tubular renal epithelium. Renal tissue has a high metabolic activity and oxygen demand, leading to enhanced exogenous toxic or mutagenic metabolites and endogenous formations of reactive oxidants metabolites. Generally speaking, the GSTM1 enzyme catalyzes the binding of these metabolites to glutathione and the subsequent conversion into mercapturic acids, making their excretion in urine or bile easier in *GSTM1* wild-type genotype subjects. However, halogenated compounds, such as trichloroethene or some pesticides, enter the mercapturic acid metabolism pathway, which are subsequently cleaved to S-(1, 2-dichlorovinyl)-L-cysteine. In the renal tubular epithelium, this compound is enriched and creates chlorothioketenes catalyzed by the renal cysteine conjugate β-lyase^26^. Even worse, chlorothioketenes are highly reactive and can form adducts with protein and DNA^27^. Therefore, an active GSTM1 enzyme forms more reactive intermediate chlorothioketenes, which can directly damage renal cells. Moreover, recent data suggests that the *GSTM1* wild-type genotype is associated with an increased risk of RCC due to involvement with the ASK1 signal transduction pathway^28^,^29^. ASK1 is a mitogen activated protein kinase (MAP3K5) that is auto-phosphorylated and activates downstream kinases, such as p38. When cells are exposed to stress, such as heat shock or reactive oxidants, ASK1 activates p38, which leads to the induction of apoptosis^28^,^29^. GSTM1 may block ASK1 by binding to ASK1 and forming a complex with the enzyme to inhibit its activity. Patients with the *GSTM1* wild-type genotype therefore may possess reduced activity of ASK1, resulting in increased risk of RCC when exposed to pollutants due to a decreased apoptotic activity response to cellular damage. Therefore, taken together, these studies provide a possible explanation for the selective nephrotoxicity and nephrocarcinogenicity of trichloroethene and pesticides in *GSTM1* wild-type subjects.

Whether the *GSTM1* and *GSTT1* polymorphisms have modified the risk of invasiveness and malignancy of RCC was also unclear until recently. A study conducted by Ahmad *et al.* found that the *GSTM1* null polymorphism might be a vital determinant of the advancement of RCC to higher clinical TNM stages^11^. In addition, Ahmad *et al.* and Salinas *et al.* found that patients with the *GSTT1* null genotype had more advanced TNM stages^11^,^14^. Therefore, we performed a meta-analysis of these studies to investigate the association between *GSTM1* and *GSTT1* polymorphisms and clinical TNM stages and Fuhrman grades of RCC. As a result, we did not find a significant association between the *GSTM1* null genotype and clinical TNM stages and Fuhrman grades of RCC (TNM stage I versus stage II + III + IV, stage I + II versus stage III + IV; Fuhrman grade 1 versus grade 2+3+4 and grade 1+2 versus grade 3+4). However, the *GSTT1* null genotype showed an association with more advanced clinical TNM stages and Fuhrman grades in RCC patients (TNM stage I + II versus stage III + IV and Fuhrman grade 1 versus grade 2+3+4). These results indicate that *GSTT1* wild-type genotype may be associated with tumors that exhibit a less invasive biological behavior and malignancy. In addition, De Martino *et al.* reported that the *GSTM1* null genotype was associated with a lower Fuhrman grade and a higher survival rate in RCC patients, while the study by Ahmad *et al.* found *GSTT1* null genotype was associated with a higher Fuhrman grade^11^,^12^. Due to the small sample size of both studies, the association between the *GSTM1* or *GSTT1* deletion variant and histological grades and survival rate of RCC remains unclear.

Several limitations should be considered in interpreting the current findings. First, reporting bias is a key threat to meta-analysis and literature-based reviews. Although publication bias was not found by funnel plots, due to small size meta-analysis, we cannot rule out the possibility that publication bias was undetected^30^. Second, even though I^2^ and τ^2^ value are very low which commonly consider as statistically acceptable, these results should be viewed with caution, since there may be still some undetected heterogeneity^31^. And, random-effects Mantel-Haenszel method for binary variable we used in the present study is hybrid and the weighting is an inverse variance but not strictly Mantel-Haenszel. Third, we didn’t conduct stratified analysis according to the publish year of included studies. It might be a bias in this meta-analysis. Fourth, the high level of environmental chemicals may accentuate or minimize the effect of the *GSTM1* and *GSTT1* wild-type genotype in oncogenesis. The interactions between genes and between genes and the environment may influence the susceptibility of RCC by more than one gene. Therefore, large-scale case control studies using microarrays, such as current genome-wide association studies (GWAS), will help to unveil potential interactions and susceptible mutations. Fifth, the sample size of studies included in our meta-analysis was not sufficiently large for a comprehensive analysis. And, nearly all of the studies were performed in Caucasian population, and additional studies are needed of other ethnic groups. Sixth, if the detail information of the included studies at an individual level were available, individual patient data meta-analysis may provide more convincing evidence.

In conclusion, our meta-analysis suggests there is an association between *GSTM1* and *GSTT1* polymorphisms and RCC. However, due to the restriction of sample size, large and well-designed studies are needed to re-evaluate these associations, especially in non-Europeans.

## Methods

### Publication search

We searched the PubMed, Institute for Scientific Information (ISI), Wangfang, Google Scholar, and Chinese National Knowledge Infrastructure (CNKI) databases for all articles describing an association between the *GSTM1* and *GSTT1* polymorphisms and RCC risk to December 2014. The key words were “*GSTM1*”, “*GSTT1*”, “renal cancer/renal cell carcinoma”, and “polymorphism/variant”. The electronic search was supplemented by checking reference lists. All of the original studies had to meet the following criteria: (1) case-control design; (2) provide sufficient data for estimating an odds ratio (OR) with a 95% confidence interval (95% CI); (3) if multiple studies were encountered that had data that overlapped by the same researchers, the most reliable studies with recently published and largest number of participants were chosen.

### Data extraction

Two of the authors (Huang and Shi) extracted all data independently following the selection criteria. The following details were collected from each study: first author’s name, publication year, country of study, ethnicity, number of cases and controls, genotyping method, and OR.

### Statistical analysis

The OR with 95% CI was used to assess the association between the *GSTM1* and *GSTT1* polymorphisms and RCC based on the genotype frequencies in cases and controls. We collected data and performed a meta-analysis of three assessments: (1) the association between the GSTM1 and GSTT1 polymorphisms and RCC; (2) the association between the GSTM1 and GSTT1 polymorphisms and RCC in studies where all cases and controls were exposed to a pesticide; and (3) the association between the GSTM1 and GSTT1 polymorphisms and RCC pathological staging. For the *GSTM1* wild-type polymorphism, the meta-analysis examined the genetic susceptibility in the three conditions described above and compared the *GSTM1* wild-type to the *GSTM1* null genotype in a recessive model (present/present+ present/null versus null/null). The same approach was used for assessment of *GSTT1*. The heterogeneity among the studies was checked with I^2^ andτ^2^ value. For the dichotomous outcomes, we used Mantel-Haenszel method and assessed this meta-analysis by using the random-effects model instead of the fixed-effects model to avoiding heterogeneity^32^,^33^,^34^,^35^,^36^. The included studies were stratified according to ethnicity for exploration of heterogeneity. Begg’s funnel plot, which is a scatter plot of effect against study size, was generated as a visual aid to detect bias or systematic heterogeneity^37^. Publication bias was assessed by Egger’s test (P < 0.05 was considered statistically significant)^38^. Meta-analysis was performed using RevMan 5.2 software (Cochrane IMS). The Begg’s funnel plot and Egger’s test were performed using STATA version 11.0 (StataCorp, College Station, Texas, USA)

## Additional Information

**How to cite this article**: Huang, W. *et al.* *GSTM1* and *GSTT1* polymorphisms contribute to renal cell carcinoma risk: evidence from an updated meta-analysis. *Sci. Rep.* **5**, 17971; doi: 10.1038/srep17971 (2015).

## Figures and Tables

**Table 1:** Main characteristics of studies on *GSTM1* and *GSTT1* polymorphisms and RCC included in the meta-analysis.

Study	Year	Country	Sample size	GSTM1					GSTT1			
				Case		Control		Sample size	Case		Control	
			(case/control)	Present	Null	Present	Null	(case/control)	Present	Null	Present	Null
Ahmad	2012	India	196/250	94	102	134	116	196/250	71	125	144	106
Salinas	2011	Spain	133/193	76	57	115	78	132/163	110	22	138	25
De Martino	2010	Austria	147/112	67	80	53	59	147/112	120	27	89	23
Wiesenhütter	2007	German	98/770	47	51	383	358	98/768	79	19	624	144
Moore	2007	Central and Eastern European	925/1247	431	424	611	555	861/1199	694	167	990	209
Buzio	2003	Italy	100/200	50	50	92	108	100/200	89	11	165	35
Sweeney	2000	American	126/505	63	63	250	255	126/505	90	36	411	94
Longuemaux	1999	French	173/211	83	88	94	116	171/210	146	25	170	40

**Table 2:** Main characteristics of studies under the conditions of all subjects with exposure to pesticides included in the meta-analysis.

Study	Year	Country	Sample size(case/control)	GSTM1				GSTT1			
				Case		Control		Case		Control	
				Present	Null	Present	Null	Present	Null	Present	Null
Karami	2008	Central and Eastern European	36/23	19	17	6	17	28	8	15	8
Buzio	2003	Italy	26/30	15	11	11	19	20	6	24	6
Brüning	1997	German	45/48	27	18	17	31	42	3	37	11

**Table 3:** Main characteristics of studies on *GSTM1* and *GSTT1* polymorphisms and clinical TNM stages of RCC included in the meta-analysis.

Study	GSTM1						GSTT1					
	I		II		III + IV		I		II		III + IV	
	Present	Null	Present	Null	Present	Null	Present	Null	Present	Null	Present	Null
Ahmad	48	22	29	31	17	49	37	33	21	39	13	53
De Martino	27	44	2	1	38	35	60	11	2	1	58	15
Sweeney	37	35	18	15	8	15	51	21	25	8	17	6
Salinas							29	30	11	9	11	25
